# Current Advances in Cheese Microbiology

**DOI:** 10.3390/foods12132577

**Published:** 2023-07-01

**Authors:** Juan A. Centeno, Javier Carballo

**Affiliations:** 1 Food Technology Area, Faculty of Sciences, University of Vigo, 32004 Ourense, Spain; jcenteno@uvigo.es; 2 Instituto de Agroecoloxía e Alimentación (IAA), University of Vigo—Campus Auga, 32004 Ourense, Spain

Cheese is a complex microbial ecosystem containing microorganisms that are either deliberately added or that enter milk, curd or cheese as contaminants. From a technological point of view, the microbiota of cheese could be classified according to the following scheme [1,2]: (i) (primary) starter cultures, composed of lactic acid bacteria (LAB) that “start” fermentation and, consequently, the acidification of milk and curd; (ii) (secondary) adjunct cultures, comprising different species of bacteria, yeasts and molds, which are intentionally added to the milk, the curd, the surface of the cheese or the interior of the previously punctured mass in the manufacture of blue cheeses, with the aim of participating in cheese ripening and the development of sensory characteristics; and (iii) (secondary) adventitious microbiota, made up of microorganisms that spontaneously contaminate milk or cheese at any of the production stages and that contribute, similarly to adjunct cultures, to the development of the characteristic appearance, texture and flavor of the different cheese varieties, yet which are occasionally responsible for defects and off-flavors.

Cheeses made from raw milk (especially from sheep or goats, or from cattle reared using extensive methods) are generally assumed to have a more intense and rich flavor than cheeses made from pasteurized or microfiltered milk. The native microbiota present in raw milk seems to be primarily responsible for the typical sensory properties and flavor development of these products. Nevertheless, it must be taken into account that the microbial diversity of raw milk (particularly raw cow’s milk) has been seriously threatened in industrialized countries as a consequence of the implementation of strict hygienic conditions on farms and during milking, as well as when stored at low temperatures. In certain current productions of traditional raw cow’s milk cheeses, the presence of adventitious bacteria such as enterococci has decreased dramatically compared to those analyzed several decades ago, and some flavor attributes are most likely being lost [3].

In order to control cheese ripening, the first and most focused approach would lie in the isolation and selection of autochthonous microbial cultures, returning them to cheesemakers for the production of the different cheese varieties. This choice would allow for the partial restoration of the flavor in productions where the use of raw milk is restricted, or where raw milk has undergone an improvement in its microbiological quality and a consequent modification of its “traditional” microbiota. The use of selected adjunct microbial cultures may enhance the typicality of the Protected Designation of Origin (PDO) cheeses, resulting in a closer sensory quality to that of traditional products [4].

In the last forty years, thousands of LAB and other microorganisms from raw milk productions, particularly in Mediterranean and southern European countries, have been isolated, characterized, tested and selected by dozens of research teams with a focus on their use in the manufacture of traditional cheeses (many of them with PDO status). However, the vast majority of implementation attempts have been unsuccessful. In our opinion, several factors have ruined much of this arduous and colossal task, among them: (i) the susceptibility of wild LAB cells (especially lactococci) to bacteriophage infections; (ii) the poor ability of many microbial strains to economically obtain cell biomass and maintain their viability and technological characteristics in dehydrated commercial preparations; and (iii) the high costs of preparing commercial cultures as freeze-dried or desiccated concentrates, which do not make the strategy profitable, given the small size of many PDO productions or the little interest of the cheesemakers.

Between the 1980s and 2000s, other technologies to control and accelerate cheese ripening were implemented, including the addition of animal or microbial enzymes and the use of attenuated or genetically modified starter cultures [5]. Attenuated LAB cultures (i.e., heat-treated *Lactobacillus helveticus*) that provide aminopeptidases with debittering activity, and which contribute to the flavor development of internally ripened hard and semi-hard cheeses, were commercially available. Likewise, adjunct culture preparations became accessible to cheesemakers, although the possibility of side effects made careful assessment advisable before their application to major production. Genetically modified LAB cultures subsequently faced legal barriers and widespread consumer disapproval.

Classically, most of the efforts in the selection of adjunct cultures for cheesemaking have focused on autochthonous LAB, but in the last decade, the attention on indigenous yeasts has increased considerably. Due to their preferential growth under aerobic conditions, these microorganisms could play a major role in the ripening of soft (smear-ripened and bloomy-rind) or semi-hard (washed-rind) surface-ripened cheese varieties, as well as in mold-ripened and fresh or short-ripened acid-curdled cheeses. Yeasts such as *Debaryomyces hansenii*, *Kluyveromyces lactis* and *Yarrowia lipolytica* have been proposed as adjunct cultures for the manufacture of various PDO cheeses [6,7].

In many cheese varieties, different species and strains of yeasts, surface bacteria, LAB and/or molds can show symbiotic effects that promote the development of organoleptic characteristics. Therefore, it is essential to investigate the interactions in these complex microbial communities to carry out an adequate selection of microbial cultures and acquire some degree of control in the microbial ecosystem of cheese. In the past, research in this area relied solely on classical microbiological techniques or culture-dependent methods, which are not suitable for handling large numbers of isolates and which fail to depict subdominant populations that can be outcompeted in vitro by more abundant microbial species. During the 1990s, the development of a range of molecular PCR-based techniques enabled the rapid identification of individual isolates at the species and strain level and led to the introduction and widespread use of culture-independent methods in most food microbiology laboratories. The implementation of these methods allowed the understanding of the complex microbial ecosystem of cheese to progress, delving into the dynamics and interactions of microbiota and its impact on cheese ripening and quality [1]. On a different matter, the development of headspace gas chromatography-olfactometry techniques made it possible to link cheese flavor chemistry directly to the selection of microbial cultures [5].

In the late 2000s, further advancement in the study of the microbial ecosystem of cheese was achieved with the development of novel culture-independent “omics” technologies that included next generation sequencing (NGS)-based methods, such as metagenomics and meta-transcriptomics targeting DNA and RNA, respectively. Next generation sequencing technologies enable the high-throughput sequencing of total microbial DNA or RNA, and thus explore the whole microbial genome without prior culturing. The combination of strain-level metagenomics with metabolomics (targeting metabolites such as volatile compounds) allows researchers to evaluate the effect of specific strains on cheese flavor. The association between the volatile compounds (volatilome) and the metagenomic clusters of the species represents a novel system for studying flavor development in cheese [8].

The multi-omics approach appears to be the best solution to correlate dynamic microbial ecology (diversity, succession and interactions within microbial communities) and metabolism in cheese making and ripening [9]. However, deciphering the cheese microbiome requires culturing strains, and a broad collection of autochthonous microorganisms should always be strongly encouraged. Currently, a combination of the results obtained by culture-dependent and “omics” methods is increasingly recommended to explore microbial communities and to profile their dynamics in cheese.

The articles included in this Special Issue show important and interesting advances and new approaches in the field of cheese microbiology. In the following section, we will briefly introduce every relevant contribution.

Microorganisms of the *Enterobacteriaceae* family are an important microbial group in cheese. They are the bacteria of fecal origin that can reach the cheese in different ways. *Enterobacteriaceae* are usually the result of the contamination of milk with fecal material due to hygienic deficiencies during milking or milk handling on farms, but they can also reach the cheese as a result of hygienic shortcomings during cheese making or the use of contaminated water [10]. The presence of *Enterobacteriaceae* in cheese, in addition to indicating poor hygienic conditions, can have various and generally negative consequences. Some species belonging to the genera *Salmonella*, *Shigella* or *Escherichia* can produce toxins and are important cheese-borne pathogens [11,12,13,14]. From a technological point of view, enterobacteria capable of fermenting lactose (coliforms) can perform undesirable fermentations when present in high counts in milk, causing a very common defect in cheese technology, known as early blowing [10,15].

In one of the papers included in this Special Issue, Hammad et al. [16] assessed the hygienic status of karish, a popular Egyptian raw-milk fresh cheese, by analyzing 200 cheese samples for total and fecal coliforms. They observed that 65% of the samples contained coliforms capable of growing at 44.5 °C. They recovered 150 thermotolerant strains that were further identified and characterized for antimicrobial susceptibility and for the production of β-lactamase and extended-spectrum β-lactamase, and subsequently performed a molecularly analysis to determine the presence of virulence and antibiotic resistance genes. Of the 150 isolated thermotolerant strains, 140 (93.3%) were identified as *Escherichia coli*. Regarded as the most outstanding result, the authors detected one Shiga toxin-producing *E. coli* strain carrying a striking virulence pattern of *stx1*−, *stx2*+, *eae*−. Eleven strains (7.8%) showed resistance to third-generation cephalosporins, and the antibiotic resistance genes *bla*SHV, *bla*CTX-M, *qnrS*, *tet*(A) and *tet*(B) were present in 4.3%, 2.8%, 0.71%, 2.1% and 0.71% of isolates, respectively. This study evidenced the poor hygienic quality of most retail karish cheeses, and its findings reinforce the need for adopting third-generation cephalosporin-resistant *E. coli* as an indicator to monitor antimicrobial resistance in raw milk cheese in order to identify potential risks to public health associated with its consumption.

As indicated above, the presence of *Enterobacteriaceae* in cheese usually demonstrates undesirable effects, but on occasion, the presence of specific species could contribute to the development of the typical sensory profile of cheeses. In relation to this, the article included in the present Issue by Ritschard et al. [17] suggests that the autochthonous species of *Enterobacteriaceae* could be a part of the typical microbiota of surface-ripened cheeses, thus participating in the development of their organoleptic properties. Ritschard et al. [17] investigated the abundance and impact of naturally occurring Gram-negative bacteria in the smear of surface-ripened cheeses. They analyzed smear samples from 15 surface-ripened semi-hard Swiss cheeses belonging to different varieties and observed an unexpectedly high number and diversity of *Proteobacteria*. *Proteus* and *Morganella* were the most dominant genera, but *Enterobacter*, *Citrobacter*, *Hafnia* and *Serratia* were also frequently found. Fourteen selected isolates were further tested for their proteolytic and lipolytic activities, and the *Proteus* isolates showed strong proteolytic activity. Finally, the authors analyzed the volatiles present on the surfaces of the cheese smears, which were known to harbor different *Proteobacteria*, and observed a volatile profile made up of compounds known to be produced by *Enterobacteriaceae* species.

*Enterococcus* is undoubtedly the most controversial genus within LAB with a harmful–helpful dual role widely described and discussed in the literature. Given their special resistance to harsh environmental conditions, enterococci are highly ubiquitous [18]. Circumscribed to their role in food in general and in cheese in particular, and due to their constant and characteristic presence in the human and mammalian intestinal tract in addition to their resistance, they were initially used as indicators of fecal contamination [19,20]. However, as the knowledge of the characteristics of this genus’ different species increased, these bacteria were assigned new roles, and new possibilities emerged with regard to their use as tools to improve various aspects of food, such as quality, safety, etc. In this Special Issue, a review article by Terzic-Vidojevic et al. [21] summarizes the current knowledge on the safety, technological properties and probiotic abilities of enterococci isolated from raw-milk cheeses, highlighting the advantages and disadvantages of their presence in cheeses. After an initial consideration of the safety aspects of the use of *Enterococcus* spp. in dairy products, the authors delve into the technological properties of the species present in traditional foods, particularly cheeses, analyzing and describing their acidifying, proteolytic and lipolytic activities, as well as their ability to produce aromatic compounds, mainly through citrate metabolism. Furthermore, many strains of enterococci isolated from dairy products have demonstrated probiotic effects, and their positive contribution to human and animal health has led some of these strains to be included in commercial probiotic products. In the last section of their article, Terzic-Vidojevic et al. [21] analyze the physiological bases and mechanisms of the probiotic action of enterococci. The authors conclude that, due to the ability of different strains to carry various virulence factors, a large amount of *in vitro* and *in vivo* testing is needed to ensure the safety and suitability of each particular strain before its use in the production of fermented foods.

Nevertheless, based on the studies carried out and the data published in this regard, it can be concluded that there are neither completely safe nor completely unsafe species of enterococci for human health, and that all their positive or negative properties are strain-specific. Thus, there are particular innocuous strains that present an outstanding metabolic profile and physiological capacities to be used in the manufacture of dairy products of superior sensory quality or with favorable probiotic effects for consumer health. Within this topic and in the next article, Lauková et al. [22] isolated and identified gelatinase-negative non-hemolytic *Enterococcus durans* strains, most of which were susceptible to commercial antibiotics. Among them, a specific strain named *E. durans* ED26E/7 exhibited particular beneficial features. It was safe, lacked genes for virulence factor such as *hyl* (hyaluronidase), *IS 16* element and *gelE* (gelatinase) and did not cause mortality in mice. Regarding its beneficial properties, this strain yielded the highest amount of β-galactosidase and also produced a bacteriocin that showed good inhibitory activity against Gram-positive bacteria (*Enterococcus avium* EA5, *Staphylococcus aureus* SA5 and *Listeria* spp., as well as many other strains of staphylococci and enterococci). The authors conclude that this is a promising strain to be used in dairy products.

Due to their glycolytic, proteolytic and lipolytic activities, along with the ability to metabolize milk citrate, microorganisms are the main players in the texture and flavor attributes of cheeses. Each cheese variety has its own and unique microbiota whose metabolic activities determine the particular characteristics of the final product. The identification of the microbial species occurring in cheese, and the establishment of the relationship between their metabolic capacities and the chemical and physical changes that take place in cheese during the ripening process have been carried out using classical methods, as previously mentioned. These firstly included the enumeration of the microbial groups using selective culture media, the isolation and identification of strains by classical biochemical methods (including, occasionally, some molecular tests) and the elucidation of their metabolic abilities. Next, these abilities were correlated with glycolytic, proteolytic and lipolytic changes, which occur during cheese ripening and which were also studied using classical instrumental techniques (electrophoresis, GC, HPLC, etc.). However, modern analytical methods allowed for a faster and more accurate identification of the cheese microbiome and a better understanding of its leadership in the organoleptic hallmark. In the last article included in this Special Issue, Anastasiou et al. [9] review these novel—and in most cases, state-of-the-art—methods that include several omics technologies (genomics, metagenomics, transcriptomics, metatranscriptomics, metaproteomics and metabolomics) along with a combination of several of these technologies (the “multi-omics” approach discussed above), describing and highlighting the many advantages associated with their use.

It is evident that, in recent years, the knowledge of cheese microbiology has expanded considerably. Nevertheless, cheese remains an ecosystem that is as complex as it is compelling. The diversity and complexity of the interactions established in cheese between microbial groups with each other and between microorganisms and various environmental factors indicate that there are still many aspects to be discovered and elucidated. Any new knowledge gathered in this area will be of significant help in addressing this scientific realm. This will allow progress in obtaining more differentiated cheeses with a much stronger personality and a more surprising sensorial property, while offering absolute microbiological safety.
